# Odor-Sight: An Interpretable Graph Neural Network Web Platform for Prediction of Odorant Activity

**DOI:** 10.1021/acs.jcim.6c01374

**Published:** 2026-08-11

**Authors:** Lucas Josue Santos Sobral, Francisco L. Feitosa, João Antônio Meletti Kunz, Pedro Koziel Diniz, Juan F. Avellaneda-Tamayo, José L. Medina-Franco, Arlindo Galvão Filho, Carolina Horta Andrade

**Affiliations:** † Laboratory for Molecular Modeling and Drug Design (LabMol), Faculdade de Farmácia, 67824 Universidade Federal de Goiás, Goiânia 74605-170, Goiás, Brazil; ‡ Advanced Knowledge Center for Immersive Technologies, Institute of Informatics, Universidade Federal de Goiás, Goiânia 74690-900, Goiás, Brazil; § Center for Research and Advancement in Fragments and Molecular Targets (CRAFT), School of Pharmaceutical Sciences at Ribeirao Preto, University of São Paulo, São Paulo 05508-060, Brazil; ∥ DIFACQUIM Research Group, Department of Pharmacy, School of Chemistry, 7180 Universidad Nacional Autónoma de México, Avenida Universidad 3000, Mexico City 04510, Mexico

## Abstract

Understanding Structure–Odor Relationships remains a significant challenge due to the intrinsic noise and subjectivity of human olfactory data. Here, we present Odor-Sight 1.0, an open-source, graph-based deep learning web platform for binary classification of odorant versus odorless molecules. Trained on a rigorously curated data set of 4,201 compounds derived from OlfactionBase, the underlying Graph Neural Network achieved a Balanced Accuracy of 0.89 ± 0.011 and a Matthews Correlation Coefficient of 0.75 ± 0.022 across 15 repeated stratified splits, demonstrating strong and reproducible performance under class imbalance. Although classical machine learning models (Random Forest, Support Vector Machine, and XGBoost) trained on Morgan fingerprints achieved comparable aggregate accuracy, only the graph-based model supports bond-level explainability and an Applicability Domain (AD) defined directly in its own learned representation. To bridge the gap between statistical learning and chemical intuition, Odor-Sight integrates EdgeSHAPer, a bond-centric Explainable AI strategy that highlights molecular substructures contributing to model predictions. Furthermore, an embedding-based AD framework is implemented to assess the prediction reliability. The platform is supported by a scalable microservices architecture, enabling accessible and high-throughput analysis. Also, case studies across four distinct odorant classes, plus a borderline odorless compound, show that the model localizes each prediction on the expected osmophore. We also benchmarked Odor-Sight against a literature platform, Odorify, using the same validation metrics. Odor-Sight provides a transparent, reproducible, and practical tool for fragrance and flavor design. The platform is freely available at https://odorsight.labmol.com.br/odorsight, and the data and scripts can be found at https://github.com/LabMolUFG/OdorSight.

## Graphical abstract

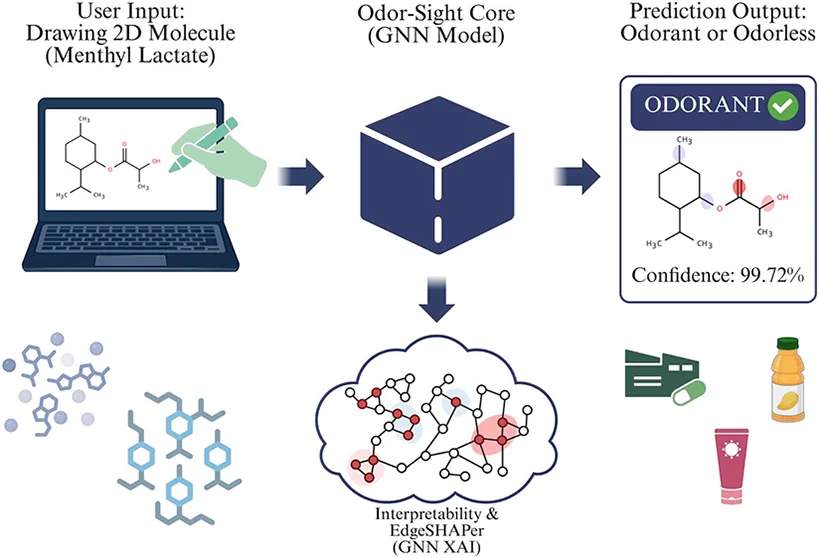

## Introduction

The prediction of olfactory properties from chemical structure—a subset of Structure–Property Associations (SPA)^1^—holds vast relevance for the food, pharmaceutical, and cosmetic industry.^2,3^ However, understanding and anticipating Structure-Odor Relationships (SOR)^4^ is computationally challenging due to the many-to-many mapping problem inherent in olfaction: structurally similar molecules can evoke distinct odors, while diverse compounds can produce similar perceptual profiles.^5,6^ Furthermore, experimental olfactory data is notoriously noisy due to the context-dependent nature of odor perception, which depends on factors such as concentration, volatility, and interindividual variability arising from genetic and environmental factors.^7^ Consequently, developing robust Quantitative Structure–Activity/Property Relationship (QSAR/QSPR) models requires methodologies that can handle noisy, imbalanced data while extracting meaningful structural patterns.^7,8^

While deep learning techniques, particularly Graph Neural Networks (GNNs), have advanced molecular representation by learning directly from molecular topology, existing odor prediction models often act as “black boxes”.^9,10^ Explainable artificial intelligence (XAI) methods are crucial in this domain to bridge the gap between statistical learning and chemical intuition. In this application note, we introduce Odor-Sight 1.0, a publicly accessible web server for binary odorant classification.

The model is trained on a curated data set derived from the OlfactionBase data set,^11^ which contains more than five thousand compounds from multiple sources. Subsequently, we apply a rigorous curation and validation protocol^12,13^ to assess model performance and integrate explainability techniques (EdgeSHAPer) to identify key molecular substructures associated with model prediction. The objective is to establish an interpretable and freely available predictive framework that not only improves classification performance for user-supplied data sets but also contributes to a deeper understanding of SOR in chemically diverse compound spaces.

## Methodology

### Data Collection

Odorant and odorless molecular data were obtained from the OlfactionBase database,^11^ an open-source web server that aggregates information on molecules associated with olfaction, from scientific literature and commercial libraries. Following extraction, the data set was manually analyzed to identify and remove all redundancies.

### Data Cleaning and Curation

Data curation followed a rigorous protocol based on the methodology of Fourches and co-workers^12,13^ implemented through an automated Python pipeline and complemented by manual inspection to ensure chemical and annotation integrity.

The initial data set comprised 5,093 compounds,^14^ including 3,969 odorants and 1,124 odorless molecules, corresponding to a class imbalance ratio of approximately 3.5:1. Entries lacking structural information or class annotations were excluded (*N* = 3). Structural validation identified and removed seven compounds with invalid SMILES representations.^15^

Salts and mixtures were subsequently processed. Salt-containing records were removed (*N* = 230). For mixture entries (*N* = 86), which predominantly consisted of the compound of interest together with its solvent, the largest molecular fragment was retained to preserve the chemically relevant structure. All tautomer SMILES were standardized. Compounds with molecular weights greater than 1,000 Da were excluded (*N* = 13), as these molecules fall outside the typical physicochemical space associated with volatile odorants. Duplicate structures were identified using canonical SMILES matching. Concordant duplicates, defined as identical structures appearing more than once with the same class label, were consolidated by retaining a single representative entry, resulting in the removal of 602 redundant records. In addition, 37 discordant duplicates, corresponding to identical structures associated with conflicting odorant and odorless annotations, were excluded from the data set to avoid introducing label ambiguity. Thus, 4,201 compounds remained after the screening process.

### Molecular Representation: Graph Features

Molecular structures were represented as graphs, where atoms correspond to nodes and bonds to edges. Node features included atom type (one-hot encoding for C, N, O, S, F, Cl, Br, I, P, and Unknown), atom degree, formal charge, hybridization, aromaticity, chirality, number of bonded hydrogens, ring membership, and normalized atomic mass. The Gasteiger partial charges were included as a continuous, atom-specific feature.^15,16^

### Edge Features Comprised Bond Type, Stereochemistry, and Conjugation

In addition, four global molecular descriptors were calculated using RDKit,^17^ including molecular weight (MolWt), octanol–water partition coefficient (Log *P*), Labute accessible surface area (LabuteASA), and molar refractivity (MolMR).^18^ These descriptors capture key physicochemical properties such as size, lipophilicity, steric effects, and polarizability, which are relevant to volatility and its receptor interactions in olfaction. These descriptors were incorporated into the node feature vectors, allowing the model to incorporate both local structural information and global physicochemical properties within the message-passing framework.

### QSAR Modeling

Model development adhered to Organization for Economic Co-operation and Development (OECD) and Tropsha QSAR guidelines.^19,20^ SMILES strings^15^ were standardized using the RDKit library^17^ to ensure consistent molecular representation. Molecular graphs were constructed using the PyTorch Geometric library,^21^ for processing by a Graph Neural Network (GNN). All computational modeling was implemented in a Python 3.10 environment.

The curated data set of 4201 compounds was partitioned into a training set (90%, *N* = 3,781) and an external test set (10%, *N* = 420) utilizing a random stratified splitting strategy. Stratification by class preserved the odorant-majority balance within each subset, preventing underrepresentation of the minority (odorless) class in the test set. To further assess the representativeness of this split, a chemical space analysis based on ECFP4 ^22^ fingerprints and t-SNE projection showed that training and test compounds occupy overlapping regions within each class, indicating that the split does not introduce systematic structural bias (Figure S1). For benchmarking purposes, we selected the Odorify platform—an artificial intelligence (AI)-driven suite utilizing deep neural networks for olfactory decoding—and verified that no compounds from our external test set overlapped with its training data set.^10^ To ensure an unbiased evaluation and to prevent information leakage, the external set was strictly held out during hyperparameter optimization.

Given the class imbalance, model performance was evaluated using Balanced Accuracy (BACC) and Matthews Correlation Coefficient (MCC), which provide reliable performance estimates under skewed class distributions.^23,24^

Hyperparameter optimization was performed using Bayesian optimization with Optuna.^25^ The search space included architectural and training parameters such as the number of GNN layers, hidden channel size, attention heads, dropout rate, learning rate, batch size, and weight decay. Each configuration was evaluated using stratified 5-fold cross-validation, with BACC as the optimization objective. Early stopping and trial pruning were applied to prevent overfitting and reduce computational cost. The best-performing configuration was retrained on the full training set and evaluated on the external test set using BACC, MCC, Precision, Recall, F1 score, and confusion matrices.

The operating threshold for binary classification was selected using the training data only, as the probability cutoff maximizing balanced accuracy on the out-of-fold predictions of the 5-fold cross-validation, and was then held fixed for the external evaluation; the external test labels were never used for threshold selection. The uncertainty of the external-test estimates was characterized in two complementary ways. First, nonparametric bootstrap 95% confidence intervals (CIs) were computed for every reported metric by resampling the external test set (*N* = 420) with replacement, stratified by class, over 10,000 iterations and taking the 2.5th and 97.5th percentiles; the threshold-independent areas under the ROC and precision–recall curves (ROC-AUC and PR-AUC) were also computed to characterize discrimination irrespective of the decision threshold, and the significance of differences relative to the benchmark model was assessed by a paired bootstrap using the same resampled molecules for both models. Second, to confirm that performance did not depend on the particular partition, the entire stratified split-and-retrain procedure was repeated for 15 independent random seeds—each drawing the held-out test set from the benchmark-disjoint subset under class stratification and retraining the model with the fixed optimized hyperparameters—and the mean, standard deviation, and range of each metric across seeds were recorded.

### Comparison with Classical Machine Learning Models

To assess the contribution of graph-based representations of GNN models relative to conventional approaches, we also trained classic Machine Learning (ML) models. Thus, Random Forest (RF), Support Vector Machine (SVM) and XGBoost (XGB) classifiers were trained based on Morgan ECFP4 fingerprints (radius 2, 2,048 bits). These baselines followed the same protocol applied to the GNN: The identical curated data set and benchmark-restricted stratified split (with matching random seeds), class weighting to address imbalance, hyperparameter tuning with Optuna under stratified 5-fold cross-validation with BACC as the objective, and an operating threshold selected solely from the training data. Robustness was characterized with the same bootstrap and repeated-split procedures, and all models were evaluated on the identical external test set.

### Explainable AI

To interpret model predictions, the EdgeSHAPer algorithm^26^ was employed to estimate bond-level contributions within the molecular graph by estimating Shapley values.^27^ This approach quantifies the marginal contribution of each edge (bond) to the model output. Unlike typical node-centric approaches, EdgeSHAPer adopts a bond-centric strategy, which is particularly relevant for chemical interpretations as it focuses on the interactions that define molecular stability and reactivity. Specifically, the algorithm utilizes Monte Carlo sampling to approximate Shapley values, enabling tractable computation despite the combinatorial complexity inherent in graph evaluations. This process allows for the identification of the “pertinent positive set”, defined as the minimal subset of chemical bonds strictly necessary for the predicted classification, thereby providing high-resolution mechanistic insights into the SOR.

### Applicability Domain

The applicability domain (AD) follows the embedding-based, model-aware framework for graph neural networks^28^ and was defined from atom-level representations at message-passing layer 3, consistent with deep-feature out-of-distribution scoring.^29^ Each atom was scored by the mean Euclidean distance to its five nearest vectors in a deterministic 25,000-atom reference bank sampled from the training set, and the molecular novelty score was the maximum atom score. To avoid self-neighbor bias, each training molecule was scored after excluding reference-bank atoms originating from that molecule. The prespecified operating boundary was the empirical 95th percentile of these leave-one-molecule-out training scores (τ_95_ = 5.296); external molecules at or below the boundary were classified as In-Domain. This explicit domain is consistent with established QSAR guidance.^19,20,30,31^ This threshold defines the domain and is derived exclusively from the training distribution (Supporting Information, Eqs S1–S2). The domain was then evaluated on the external test set of 420 compounds (308 odorants and 112 odorless, with no overlap with the training set), where domain quality was quantified by the error-detection AUC—a threshold-independent validation metric measuring how well the continuous novelty score ranks misclassified compounds ahead of correctly classified ones. As a control, the same atom-level score was additionally computed at the final pooled layer. Structural behavior was further probed with a curated panel of six canonical odorants (limonene, vanillin, menthol, benzaldehyde, eugenol, and linalool) and six structurally distant compounds (glucose, sucrose, imatinib, atorvastatin, glutathione, and sildenafil). As an orthogonal structural audit, Morgan/ECFP4 fingerprints (radius 2, 2,048 hashed bits)^22^ were compared with the training set using maximum Tanimoto similarity and an exact training-LOO p5 boundary.

### Benchmarking Against Literature Model

Model performance was benchmarked against the Odorify web server.^10^ Odorify is an AI-driven platform that utilizes deep neural networks (specifically a Transformer-CNN architecture) to predict multiple olfaction contexts, including binary odorant classification. Our external validation set (*N* = 420 compounds) was used as input for the Odorify platform. Predictions were collected and evaluated using identical metrics (BACC, MCC, Precision, Recall, and F1 score). This process allowed for an unbiased, head-to-head performance comparison between our models and the literature standard using the same held-out data.

### System Implementation and Architecture

To ensure high availability and separate user interactions from computationally intensive workflows, Odor-Sight operates on a scalable, four-tier microservices architecture (Figure S4). The User Layer features a responsive web interface built with React 19.2.0 and Next.js 16.0.5, allowing users to intuitively submit molecular structures via SMILES strings or file uploads.

Incoming requests are routed through a Gateway Layer managed by a FastAPI (v0.128.1) server, which handles validation and traffic control. To maintain system stability during high demand, requests are passed to a Redis (v6.4.0) Message Broker utilizing a priority queue system. The core predictive pipeline is executed asynchronously within the AI Worker Layer via Celery (v5.6.2). Here, SMILES strings are parsed into graph representations using RDKit (v2025.9.4), followed by inference via the optimized PyTorch Geometric (v2.7.0) GNN model. Simultaneously, the custom EdgeSHAPer engine computes bond-level contributions for interpretability, while the embedding-based Applicability Domain is evaluated from the model’s own atom-level representations using a nearest-neighbor novelty score, with Scikit-learn (v1.5.2) providing the neighbor search. Results are dynamically rendered into high-resolution visual reports using Matplotlib before being returned to the user interface.

## Results and Discussion

### Modeling

The curated data set was partitioned into 90% for training and 10% for testing, comprising 3,781 and 420 compounds, respectively, using a random stratified splitting strategy. For benchmarking purposes, it was verified that none of the compounds in the test overlapped with the training data used by the Odorify platform, as described in the Methods section.

Following hyperparameter optimization and training, model performance was validated on the external set to assess generalization to previously unseen data. The final GNN hyperparameters can be found in Section 1 “Supporting Methods and Results: Hyperparameters Selected” of the Supporting Information (SI). Given the imbalanced nature of the data set, predictive reliability was primarily evaluated using metrics appropriate to imbalanced distributions, specifically BACC, MCC, and F1 score. The optimized model demonstrated high proficiency in distinguishing odorant from odorless compounds. Across 15 repeated stratified splits, it achieved a BACC of 0.89 ± 0.011, an F1 score of 0.93 ± 0.009, and an MCC of 0.75 ± 0.022, alongside a Precision of 0.95 ± 0.011 and a Recall of 0.90 ± 0.021; discrimination was strong and threshold-independent (ROC-AUC 0.950 ± 0.008, PR-AUC 0.98 ± 0.006), as shown in Table S1. Per-metric 95% bootstrap confidence intervals on the external test set (*N* = 420) are reported in Table S2, confirming that these estimates are not an artifact of a single partition.

To assess robustness and rule out overfitting, the optimized model was also evaluated on the training set and by stratified 5-fold cross-validation (all at the 0.5 cutoff for this comparison). BACC was 0.99 in training, 0.93 ± 0.02 under cross-validation, and 0.89 on the external test set, with ROC-AUC of 1.00/0.98 ± 0.01/0.94, respectively; the classical baselines followed the same pattern. Cross-validation statistics closely tracked external-test performance across all models, indicating that the reported test metrics are representative and not an artifact of overfitting. Training, cross-validation, and test metrics for all models are reported in Table S3.

The GNN model was also compared with the baseline ML models. Across 15 repeated stratified splits, the GNN attained the highest mean value for every metric (BACC 0.89 ± 0.011, versus 0.87 for XGB, 0.86 for RF, and 0.83 for SVM). In paired bootstrap comparisons on identical test molecules, the GNN was significantly superior to the SVM (ΔBACC = 0.046; 95% CI 0.002–0.090), while its advantage over Random Forest and XGBoost was smaller and not statistically significant, with XGBoost performing on par with the GNN. All metrics are reported in Tables S4–S5.

Although the classical baselines matched the GNN on aggregate metrics, the graph-based model offers advantages that these metrics do not capture. First, its predictions are interpretable directly on the molecular graph: EdgeSHAPer estimates bond-level Shapley contributions,^26^ localizing the prediction on the specific bonds that define the osmophore, whereas feature-importance over ECFP4 bits cannot be mapped unambiguously back to substructures—a single hashed bit may encode multiple, unrelated fragments.^22^ Second, the applicability domain is defined on the model’s own learned atom-level representations rather than on external descriptors, aligning the reliability estimate with the representation that generates the prediction. Together, these properties make Odor-Sight not merely a classifier but an interpretable, domain-aware platform—capabilities that a fingerprint-based model of comparable accuracy does not provide.

### Usability

The Odor-Sight 1.0 web platform is designed for intuitive interaction and rapid deployment, requiring no local installation or programming expertise (Figure S2). Users can submit compounds for analysis through an integrated 2D chemical sketcher, by pasting SMILES strings, or via batch file uploads (CSV or SDF formats). Upon submission, the system evaluates the target and returns a binary prediction (odorant or odorless) accompanied by a probability-based confidence score.

To ensure that user-submitted molecules are processed consistently with the model’s training data, the Odor-Sight platform applies the same standardization pipeline used during curation directly on the back end. Upon submission, each structure is automatically normalized (including charge neutralization where appropriate), desalted by retaining the largest organic fragment and discarding counterions and inorganic components, and reduced to a canonical tautomer before being converted to a canonical SMILES representation for prediction. Users are not required to perform these steps manually. Additionally, if the input contains an invalid SMILES notation, such as malformed strings or broken valences, RDKit will be unable to parse the structure, resulting in an error message.

Concerning submission constraints and data privacy, Odor-Sight currently accepts one molecule per submission request. For user data safety, submitted SMILES, SDF, or MOL data are never stored on our server. To accelerate repeated queries, results are cached in Redis, keyed solely by a SHA-256 hash of the canonical SMILES, with a 7-day expiry; the original structure is never written to disk on our side.

To address the explainability of the prediction results, the user will see three images: the *Pertinent Positive Set*, the *Minimal Top-K Set*, and the *Explanations*, which together facilitate interpretation of the results, as shown in the case study below, where red areas represent structural features that favor the prediction of an odorant compound, while blue areas highlight features associated with the odorless classification.

### Applicability Domain

Following the OECD guidelines for QSAR modeling,^19^ AD was established to allow users to gauge the reliability of a given prediction. For each submitted compound, the web server reports whether it falls inside or outside this domain, allowing the reliability estimation of the corresponding prediction to be assessed. The domain was validated on the external test set. The p95 rule accepted 380 of 420 held-out compounds (90.5% coverage). Prediction error was 9.21% (35/380) inside the domain versus 15.0% (6/40) outside, corresponding to a 1.63-fold enrichment; the continuous novelty score at layer 3 ranked misclassified compounds with an error-detection AUC of 0.738. Read at the final pooled layer, the same atom-level signal fell to chance (AUC 0.51), confirming that the structural information underlying novelty detection resides in the intermediate representation rather than in the pooled output. All six canonical odorants were retained inside the domain, while two of the six structurally distant compounds, glutathione and sildenafil, were flagged as out-of-domain (Table S6). Notably, whole-molecule fingerprint similarity and atom-level novelty can diverge—imatinib, for instance, is globally dissimilar to the training set by ECFP4 yet remains inside the domain because it is atom-locally familiar—underscoring that the domain reflects the model’s learned representation rather than bulk structural similarity (Supporting Information, Figure S3 and Orthogonal structural audit). The two-dimensional domain map is provided in the Supporting Information (Figure S3).

Descriptor-space domains admit molecules the model handles poorly and reject molecules it handles well, because their notion of similarity is independent of the model.^30,31^ Grounding the domain in the model’s own learned embedding removes this mismatch;^28,29^ as the fingerprint audit above illustrates for imatinib, which is globally dissimilar by ECFP4 yet atom-locally familiar and correctly handled. Critically, the domain boundary itself (τ95) is derived independently from the training data alone, using none of the tuned parameters from the source framework, and its validity is confirmed empirically here: The comparison above shows that which internal representation is used is decisive, not merely whether the domain is model-derived.

It should be emphasized that the training data is dominated by cosmetic and fragrance-related chemicals. Consequently, the reliable prediction space of Odor-Sight is centered on this chemical domain, and compounds from chemical classes underrepresented in the training set are more likely to fall outside the AD. For these compounds, the AD flag will typically signal extrapolation, and predictions should be interpreted with caution. We therefore recommend that end users always consult the AD status alongside the predicted label, treating out-of-domain results as exploratory rather than definitive.

### Case Study: Interpretable Odorant Classification

To illustrate the predictive and interpretive capabilities of Odor-Sight 1.0, we evaluated odorant compounds from several distinct chemical classes. We also selected an odorless compound located close to the model’s decision boundary to demonstrate interpretability in a borderline case. All molecules used in this study were verified to be absent from the training data set, and all of them were classified as “In domain,” with a Layer-3 atom novelty value below the threshold of 5.296 as was described in the Methodology section.

The first molecule analyzed was (5-methyl-2-propan-2-ylcyclohexyl)­2-hydroxypropanoate, commercially known as menthyl lactate, as shown in Figure 1. Three parameters determine the interpretability of the prediction. Figure 1A: Bond Importance, shows the impact that each bond in the molecule has on the prediction. Figure 1B: Minimal Top K Set, shows the bonds that contributed most to the molecule’s prediction. Figure 1C: Pertinent Positive Set, indicates the minimum number of bonds required to maintain that molecule’s prediction status, the essential ones. The explainability figures for the other molecules can be found in the SM, along with the applicability domain for each one (Figures S5–S13).

**Figure 1:**
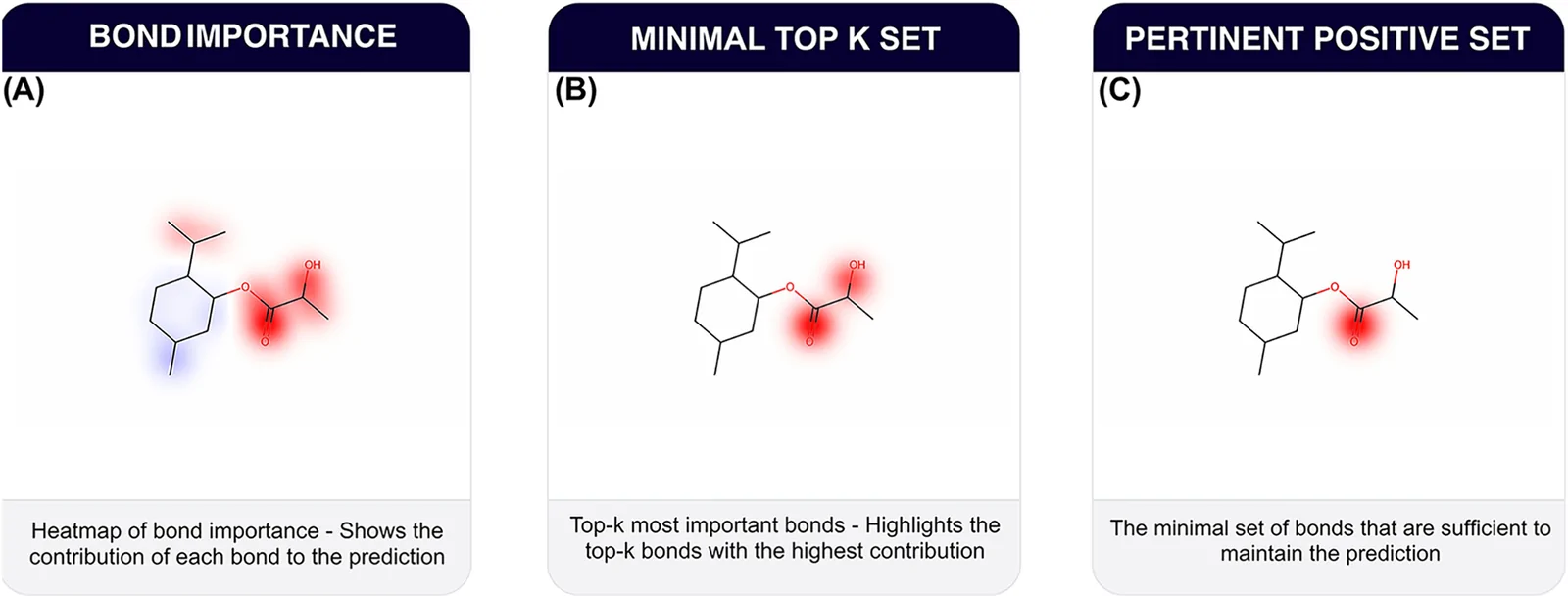
Illustration of the results generated by the Odor-Sight 1.0 Explainable AI module for (5-methyl-2-propan-2-ylcyclohexyl)­2-hydroxypropanoate (Menthyl Lactate). Red regions indicate a positive contribution toward the odorant prediction, whereas blue regions correspond to contributions toward the odorless prediction.

The menthyl lactate shows a prediction confidence of 97.94%, is a well-documented molecule in literature, featuring a faint odor reminiscent of tobacco and chamomile.^32^ Both Minimal Top K set and Pertinent Positive Set isolated the ester moiety (C­(=O)­O) as the primary structural determinant, aligning with established SOR where esters drive fruity/floral profiles.^33^ The heatmap visualization further highlights that while the ester contributes strongly positively (red), peripheral aliphatic regions display minor negative contributions (blue). It is crucial to note that in olfaction, these negative regions do not represent intrinsically “odorless” substructures, as perception relies on the combinatorial interaction of the entire molecule with olfactory receptors.^34^

As for the other molecules (all of them with 100% confidence prediction), we analyzed an aliphatic thiol, Nonane-1-thiol; its Pertinent Positive Set accurately concentrated on the sulfhydryl (−SH) group, consistent with its well-documented role as a potent osmophore in sulfurous odorants.^35,36^ Similarly, for the acyclic monoterpene alcohol, (2*E*)-3,7-dimethylocta-2,6-dien-1-ol, commercially known as geraniol, EdgeSHAPer highlighted the primary hydroxyl (−OH) group and its adjacent double bond (with a high positive contribution) as the Minimal Top-K Set, a structural motif classic to floral scent profiles.^1^ Crucially, the model’s ability to learn beyond heteroatoms was validated using 1-methyl-4-(prop-1-en-2-yl)­cyclohex-1-ene, known as limonene. Lacking oxygen or sulfur, the Pertinent Positive Set correctly isolated the exocyclic double bond as the primary structural driver for its prediction, aligning with established SOR for terpene hydrocarbons.^35,37^

Conversely, we challenged the model with a bulky menthyl half-ester derivative, 4-[(1*R*,2*S*,5*R*)-5-methyl-2-propan-2-ylcyclohexyl]­oxy-4-oxobutanoic acid, also known as monomenthyl succinate. Odor-Sight correctly classified it as odorless, but with a borderline prediction confidence of 69.38% (Layer-3 novelty: 3.697). The EdgeSHAPer analysis reflects this physicochemical uncertainty: rather than a concentrated positive osmophore, the heatmap displays diffuse negative (blue) contributions distributed across the aliphatic scaffold and the terminal carboxylic acid. This borderline output chemically translates to the molecule’s conflicting features; while the ester moiety might weakly trigger odorant receptors, the overall steric bulk—approaching the ∼300 Da upper limit for olfaction—and the strong hydrogen-bonding capacity of the carboxylic acid group can drastically reduce vapor pressure, ultimately pushing the compound just over the decision boundary into the odorless space.^35,38^

### Benchmarking and Comparative Analysis

To contextualize the predictive power of Odor-Sight 1.0 against current state-of-the-art platforms, we performed a comparative analysis against the Odorify web server.^10^ To ensure a fair, head-to-head evaluation, Odorify was tested using our strictly held-out external validation set (*N* = 420), verifying beforehand that no overlap existed with Odorify’s training data.

While both platforms employ deep learning, they utilize distinct architectures: Odor-Sight utilizes a GNN, whereas Odorify employs a Transformer-CNN framework. On the external set, Odorify achieved a BACC of 0.693, an MCC of 0.527, a Precision of 0.819, a Recall of 0.984, and an F1 score of 0.894. In contrast, Odor-Sight demonstrated superior overall robustness: BACC 0.886, MCC 0.745, Precision 0.950, Recall 0.904, and F1 score 0.926. The substantial improvements in MCC (+0.22) and BACC (+0.19) are particularly significant. Because olfactory data sets are intrinsically imbalanced, these metrics confirm that Odor-Sight is highly effective at minimizing false positives and correctly modeling the minority class without sacrificing overall accuracy.^23^ Furthermore, while both tools offer explainability, Odor-Sight’s bond-centric EdgeSHAPer methodology provides a highly granular, synthetically relevant interpretation compared to Odorify’s feature-based Layer-wise Relevance Propagation.

## Conclusion

The Odor-Sight 1.0 platform provides an accessible and interpretable computational solution for distinguishing between odorant and odorless molecules. By integrating an optimized GNN with bond-centric explainable AI (EdgeSHAPer) and an embedding-based AD, the platform demonstrates strong predictive performance on an imbalanced olfactory data set while offering structural insights into model predictions.

Furthermore, the intuitive web interface requires no programming expertise, enabling straightforward deployment for researchers and industry professionals and supporting both individual and batch processing. In addition, the integration of explainability and AD assessment provides users with tools to interpret predictions and evaluate their reliability within the modeled chemical space.

We acknowledge that the reduction of the multifaceted nature of human olfactory perception to a binary classification represents a necessary modeling simplification. This approach cannot fully capture complex biological complexities, including concentration-dependent perception and interindividual variability driven by genetic and environmental factors. In addition, the curated odorless subset (more than 600 compounds) remains smaller than the odorant class and is dominated by cosmetic and fragrance-related chemicals, so it may underrepresent other chemical spaces such as industrial chemicals and agrochemicals; predictions for such compounds should be interpreted alongside the Applicability Domain assessment rather than assumed to generalize.

Future iterations of Odor-Sight will prioritize the expansion of predictive capabilities to include more detailed representations of olfactory function, including olfactory receptor (OR) mapping and odorant-OR interaction analysis.

Ultimately, Odor-Sight serves as a transparent and reliable open-source resource to accelerate candidate screening and optimize fragrance and flavors. The web platform is freely accessible at https://odorsight.labmol.com.br/odorsight.

## Supplementary Material



## Data Availability

The curated data sets (containing molecular structures and binary labels) used for model training and external validation are provided in the Supporting Information. The Python source code, including workflows for data preprocessing, descriptor calculation, data partitioning, model training, and evaluation, is open-source and freely available on GitHub at https://github.com/LabMolUFG/OdorSight.

## Notes

Selected hyperparameters, chemical space analysis, applicability domain definition and validation, complete performance tables for all models, system architecture diagrams, and the EdgeSHAPer explanations for the case-study compounds (PDF)
