# Supplementary material for: Odor-Sight: An Interpretable Graph Neural Network Web Platform for Prediction of Odorant Activity
Source: J Chem Inf Model. 2026 Aug 11;66(16):9721–8. doi: 10.1021/acs.jcim.6c01374 (PMC13508998; doi:10.1021/acs.jcim.6c01374)
Supplement: Supplementary file 1 [file ci6c01374_si_001.pdf]

Supporting Information

Odor-Sight: An Interpretable Graph Neural Network Web Platform for Prediction of Odorant Activity

*Lucas Josue Santos Sobral[1-3]; Francisco Lucas Feitosa[1-3]; João Antônio Meletti Kunz[1-2]; Pedro Koziel Diniz[1-2]; Juan F. Avellaneda-Tamayo[4], José L. Medina-Franco[4], Arlindo Galvão Filho[2]; Carolina Horta Andrade[1-3]**

[1]Laboratory for Molecular Modeling and Drug Design (LabMol), Faculdade de Farmácia, Universidade Federal de Goiás, Goiânia, Goiás, Brazil.

[2]Advanced Knowledge Center for Immersive Technologies, Institute of Informatics, Universidade Federal de Goiás, Goiânia, 74605-170, GO, Brazil.

[3] Center for Research and Advancement in Fragments and Molecular Targets (CRAFT), School of Pharmaceutical Sciences at Ribeirao Preto, University of São Paulo, São Paulo 05508-060, Brazil.

[4] DIFACQUIM Research Group, Department of Pharmacy, School of Chemistry, Universidad Nacional Autónoma de México, Avenida Universidad 3000, Mexico City 04510, Mexico.

Corresponding Author

*Address for correspondence: Laboratory for Molecular Modeling and Design, Faculdade de Farmácia, Universidade Federal de Goiás, Goiânia, GO, 74605-170, Brazil; Telephone: +55 62 3209-6451; E-mail: carolina@ufg.br

Table of Contents

1. Supplementary Methods and Results: Hyperparameters Selected......................2
2. Chemical space analysis..3
3. Applicability Domain: novelty score and domain boundary 4
4. GNN Performance in 15 repeated stratified splits 5
5. GNN 95% bootstrap confidence intervals on the external test set 6
6. Training, cross-validation and test metrics for all models (0.5 cutoff) 7
7. Performance GNN vs classical ML models 8
8. Paired bootstrap comparison of GNN and classical ML models 8
9. Odor-Sight Overview Platform...9
10. Applicability domain: reference panel and structural audit 10
11. Odor-Sight applicability domain map 12
12. Odor-Sight System Architecture 12
13. Odor-Sight case studies...13

1. Supplementary Methods and Results: Hyperparameters Selected

GNN Model

The hyperparameters of the Graph Neural Network (GNN) model were optimized during the training phase to maximize predictive performance while avoiding overfitting. The optimization procedure explored different configurations of architectural and training parameters, including the number of layers, hidden feature dimensions, attention heads, dropout rate, learning rate, batch size, and weight decay.

The final model architecture corresponds to a Graph Attention Network (GAT) with six graph convolution layers and a hidden dimension of 256 channels. The attention mechanism uses two heads per layer, and regularization is applied through dropout and weight decay. The training procedure was performed using a batch size of 32 and the Adam optimizer with a learning rate of 2.87×10^{-4}.

The final hyperparameter configuration used in the model is summarized below.

```
{"model_type": "GAT",
 "num_layers": 6,
 "hidden_channels": 256,
 "heads": 2,
 "dropout": 0.21,
 "lr": 2.87e-04,
 "batch_size": 32,
 "weight_decay": 2.62e-04}
```

2. Chemical space analysis

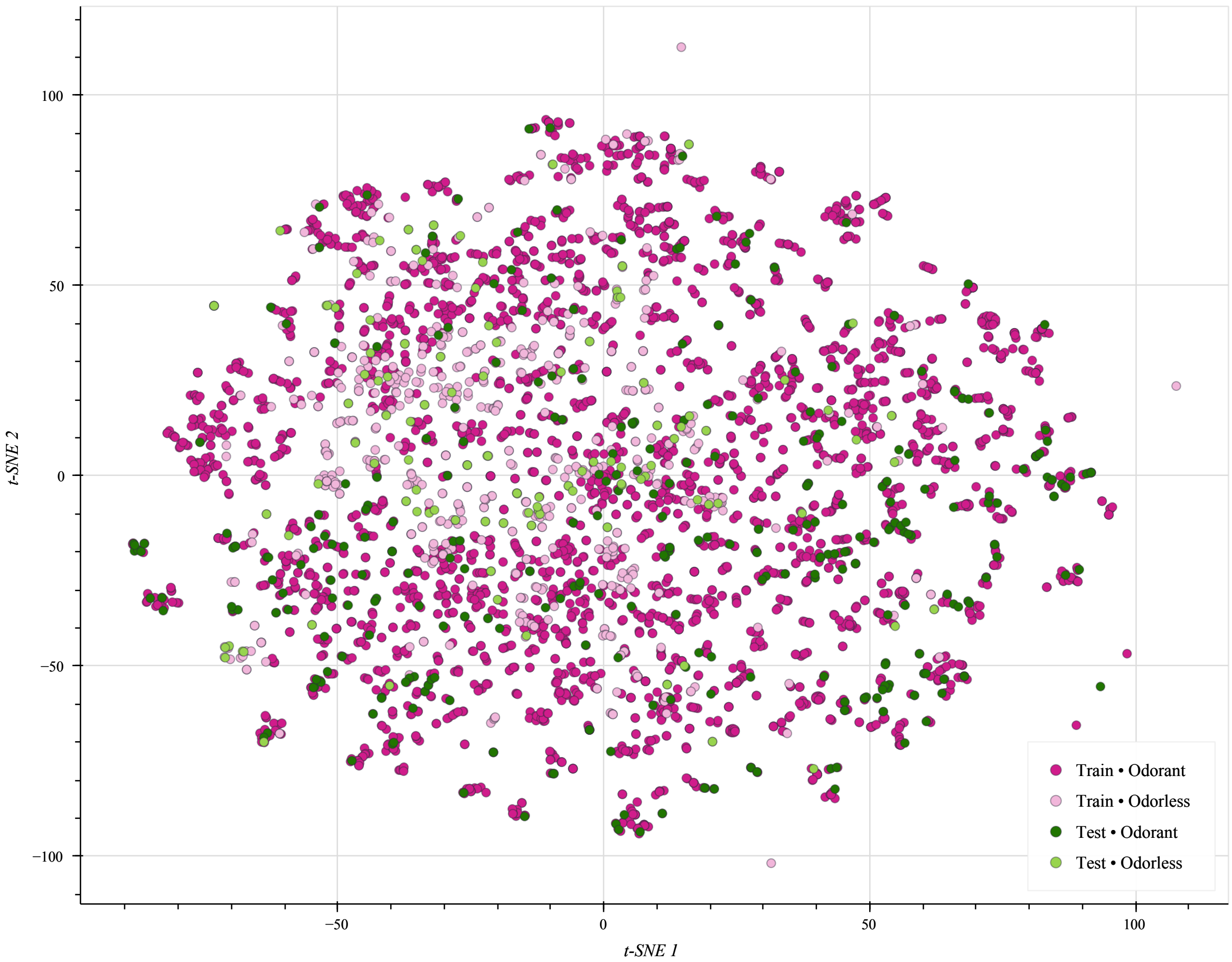

Figure S1 - Visual representation of the distribution of the training and test sets in chemical space, colored by odorant/odorless classification.

To generate the visual representation molecular structures were encoded as ECFP4 (Morgan, radius 2) fingerprints and projected into two dimensions using t-distributed stochastic neighbor embedding (t-SNE). Each point represents a compound, colored according to one of four categories: training set odorant compounds, training set odorless compounds, test set odorant compounds, and test set odorless compounds. The overlapping distribution of training and test compounds within each class indicates that the test set falls within the structural space covered by the training data, supporting the absence of systematic structural bias introduced by the data split. An interactive HTML version of this map, allowing visualization of each compound's chemical structure, is available in the associated GitHub repository https://github.com/LabMolUFG/OdorSight.

3. Applicability Domain: novelty score and domain boundary

Atom-level novelty score

Each molecule is passed through the trained GNN. At message-passing layer 3, every atom a carries a learned embedding $\mathbf{h}_a \in \mathbb{R}^{256}$ encoding its local chemical environment. Each atom is compared with its five nearest vectors in a frozen reference bank of training-set atom embeddings, and its atom score is the mean of those five Euclidean distances. The molecular novelty score is the maximum atom score:

$$novelty(mol) = max_{a \in atoms} \frac{1}{k} \sum_{j=1}^{k} \left|\left| h_a - h_a^{(j)} \right|\right| \qquad k = 5 \quad \text{(S1)}$$

where $\mathbf{h}_a^{(j)}$ is the j-th nearest training-atom embedding to atom a and $\|\cdot\|$ is the Euclidean distance. The maximum over atoms ensures that a single unfamiliar atom environment is sufficient to raise the score.

Domain boundary

A molecule is classified as in-domain when its novelty score does not exceed the threshold τ_{95}, defined as the 95th percentile of the molecule-level leave-one-out novelty distribution of the training set:

$$In\ domain \leftrightarrow novelty\ (mol) \leq \tau_{95}, \qquad \tau_{95} = 5.296 \quad \text{(S2)}$$

For calibration, each training molecule is scored after removing every bank vector originating from that molecule, so that the threshold derives exclusively from the training distribution and uses no test-set information.

4. GNN Performance in 15 repeated stratified splits

Table S1 - Predictive performance of the Odor-Sight GNN across 15 independent repeated stratified splits. For each split, the dataset was partitioned into training and external test subsets (90/10, stratified by class; test set N = 420) and the model was retrained from scratch using the fixed optimized hyperparameters (Section 1 - Supplementary Methods and Results: Hyperparameters Selected). The operating threshold was selected on the training data only, never on the external test set. Values are reported as mean ± standard deviation, together with the observed minimum–maximum across the 15 splits.

Metric	Mean ± SD	Min-Max
**BACC**	0.89 ± 0.011	0.87–0.91
**MCC**	0.75 ± 0.022	0.70–0.79
**Precision**	0.95 ± 0.011	0.93–0.97
**Recall**	0.90 ± 0.021	0.87–0.94
**Specificity**	0.87 ± 0.032	0.82–0.92
**F1**	0.93 ± 0.009	0.91–0.94
**ROC-AUC**	0.95 ± 0.008	0.94–0.96
**PR-AUC**	0.98 ± 0.006	0.97–0.99

5. GNN 95% bootstrap confidence intervals on the external test set

Table S2 - Bootstrap 95% confidence intervals for the Odor-Sight GNN on the external test set (N = 420). Confidence intervals were obtained from 10,000 stratified bootstrap resamples (with replacement) of the external test set, recomputing every metric on each resample; the reported interval spans the 2.5th–97.5th percentiles of the resulting distribution. Metrics are shown at the operating threshold derived exclusively from the training data by 5-fold cross-validation (0.944), ensuring no information from the test set influenced threshold selection.

Metric	Point	95% CI
**BACC**	0.89	0.86–0.92
**MCC**	0.72	0.65–0.78
**Precision**	0.97	0.95–0.99
**Recall**	0.85	0.81–0.89
**Specificity**	0.93	0.88–0.97
**F1**	0.91	0.88–0.93
**ROC-AUC**	0.94	0.91–0.97
**PR-AUC**	0.97	0.95–0.99

6. Training, cross-validation and test metrics for all models (0.5 cutoff)

Table S3 Training, cross-validation, and external test performance of the Odor-Sight GNN and the classical ML baselines. Each cell reports train / CV / test values; cross-validation values are the mean ± standard deviation across stratified 5-fold splits of the training set. All threshold-dependent metrics were computed at the 0.5 cutoff.

Model	BACC	MCC	F1	ROC-AUC	PR-AUC
**GNN**	0.99 / 0.93 ± 0.02 / 0.89	0.93 / 0.82 ± 0.03 / 0.75	0.99 / 0.97 ± <0.01 / 0.93	1.00 / 0.98 ± 0.01 / 0.94	1.00 / 0.99 ± <0.01 / 0.97
**RF**	0.96 / 0.89 ± 0.01 / 0.85	0.88 / 0.75 ± 0.03 / 0.71	0.98 / 0.96 ± <0.01 / 0.92	1.00 / 0.96 ± 0.01 / 0.92	1.00 / 0.99 ± <0.01 / 0.96
**SVM**	0.97 / 0.84 ± 0.02 / 0.84	0.95 / 0.68 ± 0.03 / 0.69	0.99 / 0.95 ± <0.01 / 0.92	0.99 / 0.93 ± 0.01 / 0.92	1.00 / 0.98 ± <0.01 / 0.97
**XGB**	0.97 / 0.90 ± 0.01 / 0.88	0.89 / 0.76 ± 0.03 / 0.75	0.98 / 0.96 ± <0.01 / 0.93	0.99 / 0.97 ± 0.01 / 0.94	1.00 / 0.99 ± <0.01 / 0.97

7. Performance GNN vs classical ML models

Table S4 - Per-metric performance of the Odor-Sight GNN and the classical ML baselines (Random Forest, SVM, and XGBoost on Morgan/ECFP4 fingerprints) across 15 repeated stratified splits, reported as mean ± standard deviation.

Metric	GNN (GAT)	RF (ECFP4)	SVM (ECFP4)	XGBoost (ECFP4)
**BACC**	0.89 ± 0.011	0.86 ± 0.011	0.83 ± 0.028	0.87 ± 0.012
**MCC**	0.75 ± 0.022	0.70 ± 0.027	0.62 ± 0.063	0.71 ± 0.021
**Precision**	0.95 ± 0.011	0.94 ± 0.009	0.94 ± 0.017	0.94 ± 0.013
**Recall**	0.90 ± 0.021	0.89 ± 0.025	0.82 ± 0.068	0.90 ± 0.027
**Specificity**	0.87 ± 0.032	0.83 ± 0.028	0.84 ± 0.051	0.84 ± 0.040
**F1**	0.93 ± 0.009	0.91 ± 0.011	0.87 ± 0.038	0.92 ± 0.011
**ROC-AUC**	0.95 ± 0.008	0.93 ± 0.011	0.91 ± 0.015	0.94 ± 0.008
**PR-AUC**	0.98 ± 0.006	0.97 ± 0.006	0.96 ± 0.009	0.98 ± 0.005

8. Paired bootstrap comparison of GNN and classical ML models

Table S5 - Paired bootstrap comparison of each classical baseline against the Odor-Sight GNN on identical external-test molecules. Δ = baseline − GNN (negative values favor the GNN); 95% CI in parentheses; * = CI excludes zero.

Δ = baseline − GNN	RF (ECFP4)	SVM (ECFP4)	XGBoost (ECFP4)
**BACC**	−0.035<br>(−0.076, 0.004)	−0.046*<br>(−0.090, −0.002)	−0.010<br>(−0.044, 0.025)
**MCC**	−0.040<br>(−0.116, 0.033)	−0.059<br>(−0.144, 0.024)	+0.005<br>(−0.056, 0.067)
**Precision**	−0.030*<br>(−0.056, −0.005)	−0.036*<br>(−0.064, −0.009)	−0.014<br>(−0.037, 0.008)
**Recall**	+0.019<br>(−0.010, 0.052)	+0.016<br>(−0.019, 0.052)	+0.026<br>(0.000, 0.052)
**Specificity**	−0.089*<br>(−0.161, −0.018)	−0.107*<br>(−0.188, −0.027)	−0.045<br>(−0.107, 0.018)
**F1**	−0.005<br>(−0.026, 0.016)	−0.009<br>(−0.034, 0.015)	+0.006<br>(−0.011, 0.024)
**ROC-AUC**	−0.018<br>(−0.048, 0.010)	−0.020<br>(−0.048, 0.010)	−0.004<br>(−0.034, 0.026)
**PR-AUC**	−0.008<br>(−0.028, 0.012)	−0.006<br>(−0.023, 0.015)	−0.003<br>(−0.027, 0.020)

9. Odor-Sight Overview Platform

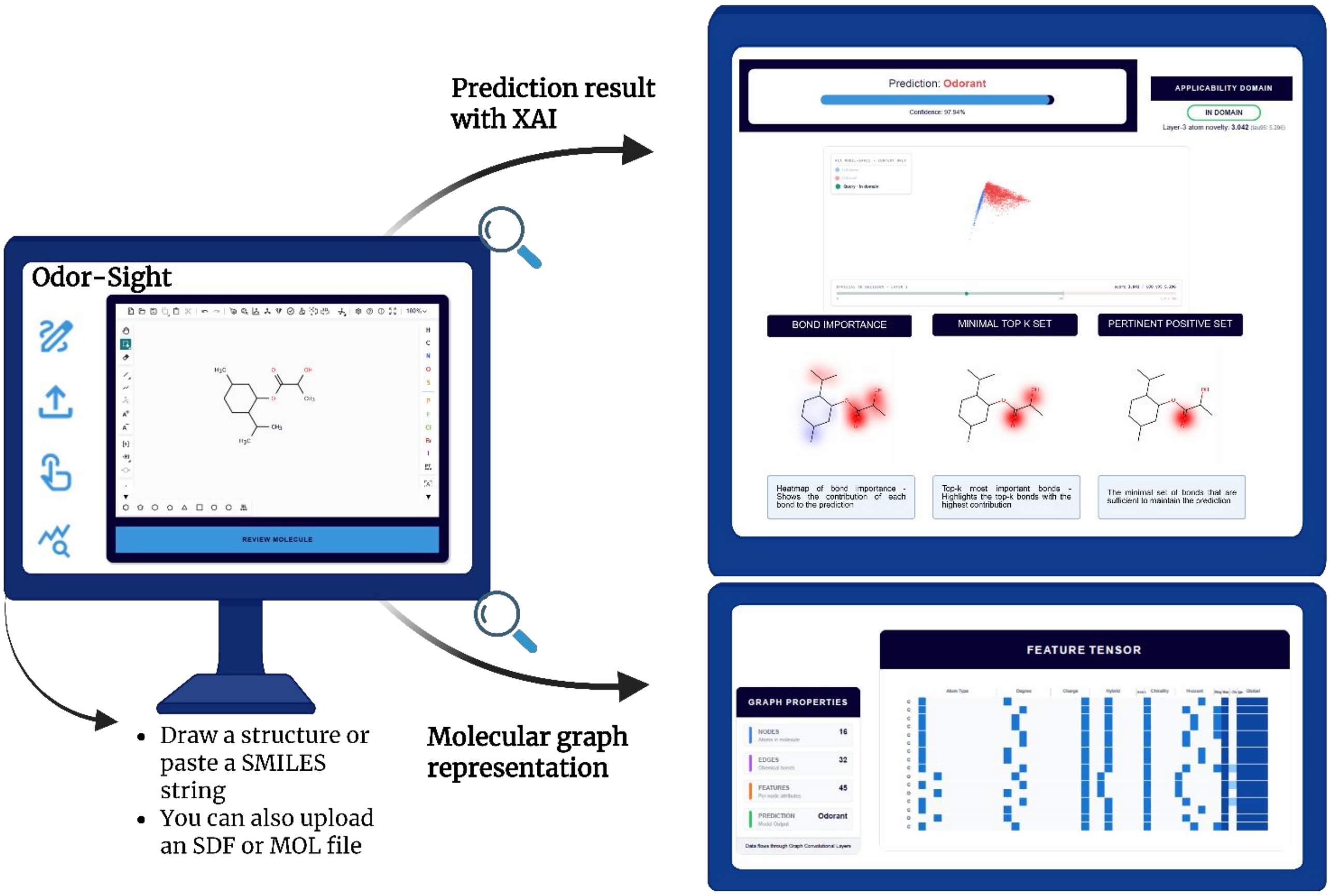

Figure S2 - Overview of the Odor-Sight 1.0 web platform workflow and user interface. The platform allows users to submit molecules via a drawn structure, SMILES string, or batch file upload (left). The system processes the input, standardizes it, and returns a comprehensive, interpretable output (right), including binary odor prediction, confidence score, XAI visualizations (bond importance and pertinent positive sets), applicability domain status, and molecular graph information.

10. Applicability domain: reference panel and structural audit

Table S6 - Layer-3 atom novelty scores for the twelve reference compounds, relative to the training boundary τ_{95} = 5.296. Compounds are ordered by increasing novelty within each panel group.

Molecule	Panel group	Novelty score	Status
**Benzaldehyde**	Canonical odorant	2.31	In-domain
**Eugenol**	Canonical odorant	2.38	In-domain
**Menthol**	Canonical odorant	2.83	In-domain
**Vanillin**	Canonical odorant	3.26	In-domain
**Linalool**	Canonical odorant	3.96	In-domain
**Limonene**	Canonical odorant	4.55	In-domain
**Sucrose**	Structurally distant	2.49	In-domain
**Glucose**	Structurally distant	2.94	In-domain
**Imatinib**	Structurally distant	5.04	In-domain
**Atorvastatin**	Structurally distant	5.23	In-domain
**Sildenafil**	Structurally distant	5.70	Out-of-domain
**Glutathione**	Structurally distant	5.83	Out-of-domain

Orthogonal structural audit (ECFP4)

To interpret the domain decisions from an independent angle, each reference compound was also compared with the training set by whole-molecule structure, using the maximum Tanimoto similarity of ECFP4 fingerprints against a training-set boundary (the 5th percentile of training similarities, 0.318). This descriptor-based view and the atom-level novelty score answer different questions, and their disagreements are informative. Imatinib is globally dissimilar to the training set by ECFP4 — its maximum training similarity (0.240) falls below the boundary and its scaffold is absent — yet none of its hashed ECFP4 substructures is unseen in training,

and its atom-level score remains just inside the domain (5.043 versus τ_{95} = 5.296). Glutathione shows the opposite pattern: it lies within the ECFP4 similarity boundary (0.463) but is flagged out-of-domain by the atom-level score, a rejection associated with its unusually polar, hydrogen-bond-rich local environments rather than with low whole-molecule similarity. These fingerprints serve as an orthogonal audit that explains such disagreements; they do not override the adopted atom-level decision. In both directions, this confirms that domain membership is governed by the learned atom-level representation, not by whole-molecule structural similarity.

11. Odor-Sight applicability domain map

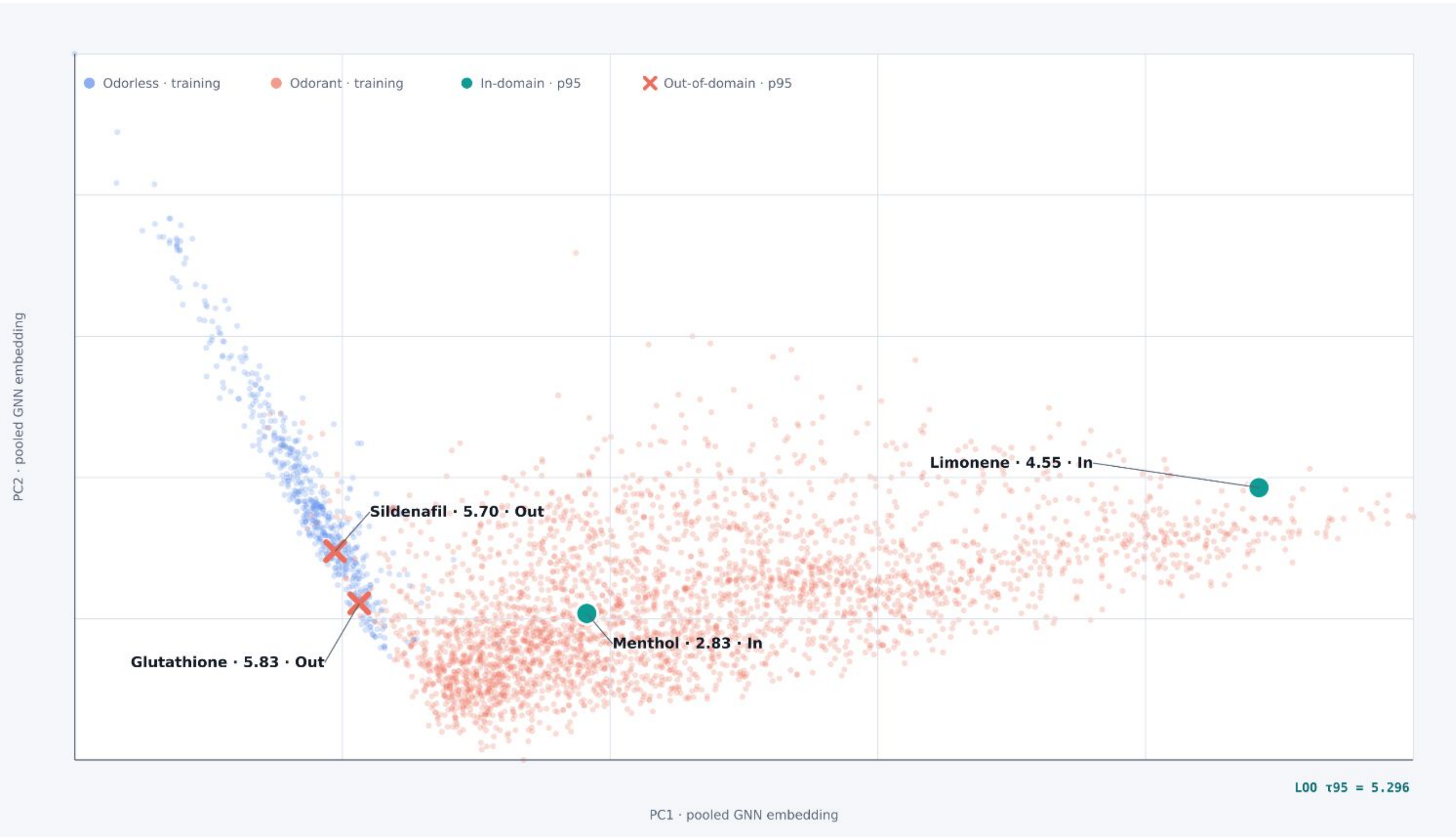

Figure S3 - Applicability domain map. Training compounds projected onto the two leading principal components of the pooled GNN embedding, colored by class (odorant, odorless). The two-dimensional projection is contextual only; in-domain/out-of-domain status is assigned solely from the atom-level novelty score (τ_{95} = 5.296), not from position on the map. Canonical odorants (e.g., limonene, novelty = 4.55; menthol, novelty = 2.83) fall inside the domain, whereas sildenafil (5.70) and glutathione (5.83) exceed the threshold and are flagged out-of-domain despite projecting within the populated region of the map, illustrating that the decision derives from atom-level structure rather than from the projected coordinates.

12. Odor-Sight System Architecture

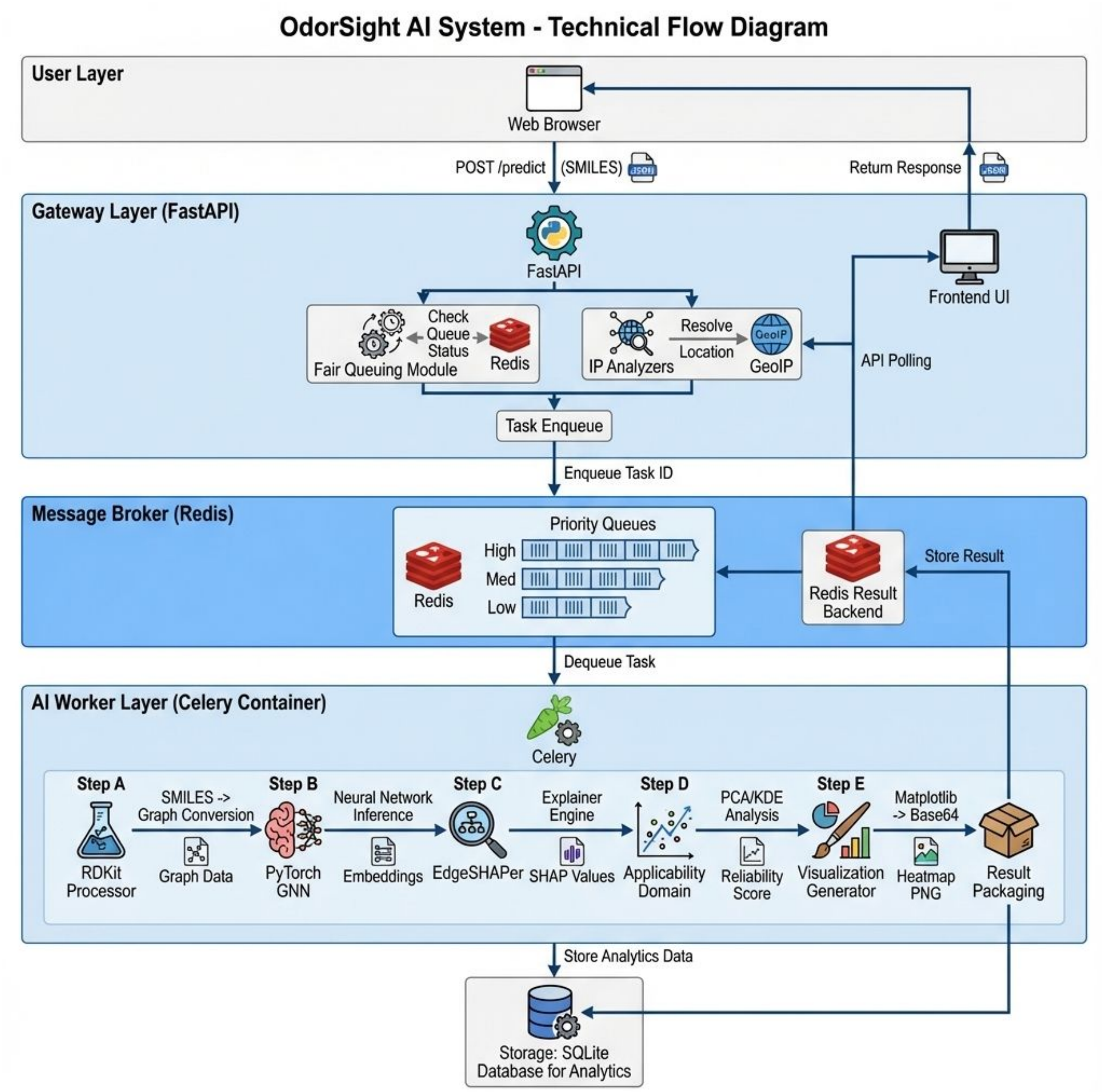

Figure S4 – OdorSight AI System – Technical Flow Diagram. Overview of the four-layer microservices architecture of the OdorSight platform. User requests submitted via a web interface (SMILES input) are handled by a FastAPI gateway and dispatched to a Redis message broker with priority queues. Celery workers execute the prediction pipeline, including SMILES-to-graph conversion with RDKit, inference using a PyTorch-based GNN, explainability analysis with EdgeSHAPer, applicability domain assessment via PCA/KDE, and visualization generation. Analytics data are stored in a SQLite database.

13. Odor-Sight case studies

For each compound analyzed in the case studies, the Odor-Sight platform generates two complementary outputs, reproduced in this section. The first is the EdgeSHAPer explanation, comprising the pertinent positive set, the minimal top-K set, and the full bond-importance heatmap. Red indicates bonds contributing toward the odorant class and blue toward the odorless class.

The second is the applicability domain assessment, which reports the layer-3 atom novelty score of the query molecule against the training boundary $\tau_{95} = 5.296$, together with a two-dimensional projection of the training set provided for visual context only. The query molecule (green) is projected onto the two leading principal components of the pooled GNN embedding alongside the training set (odorant, red; odorless, blue). Values at or below the boundary are classified as in-domain.

Menthyl lactate

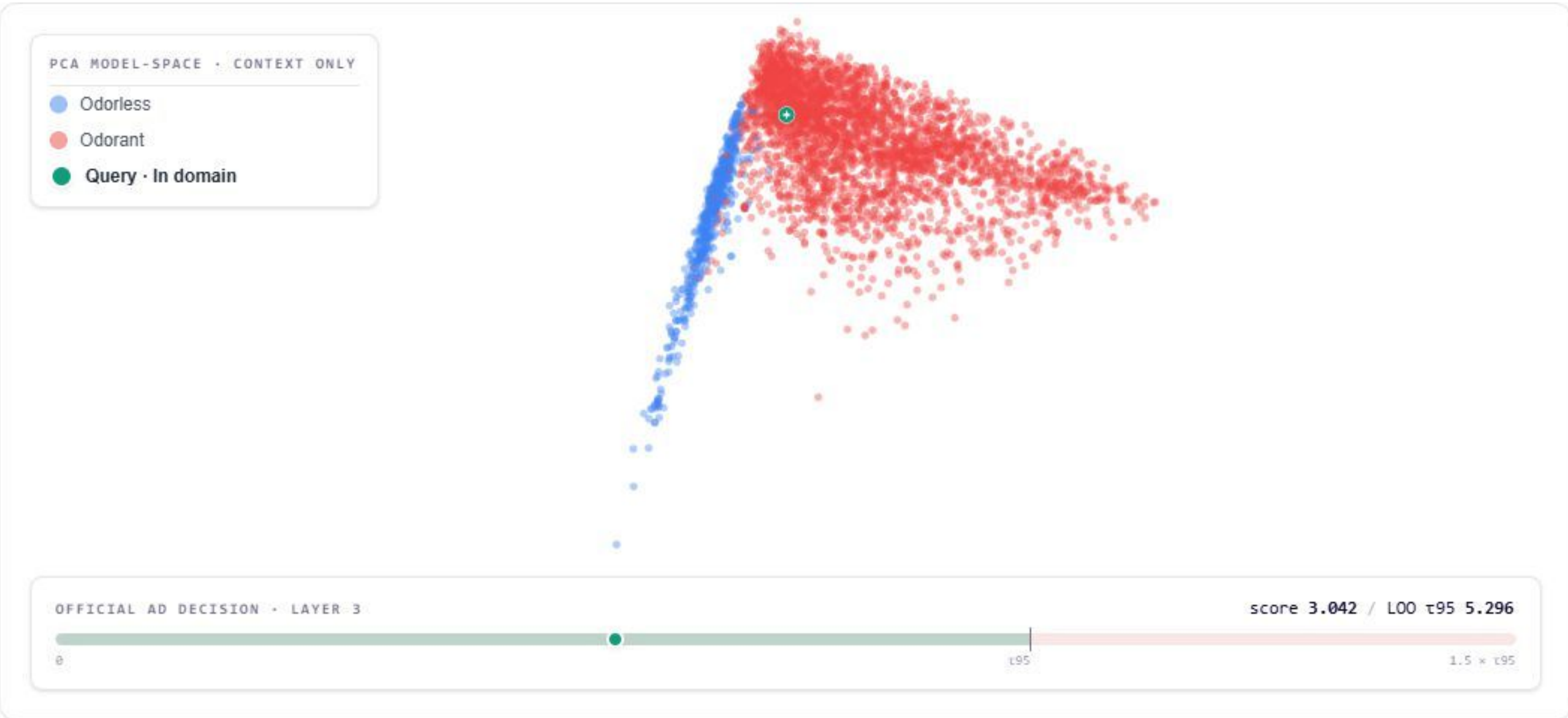

Figure S5 - Applicability domain assessment for menthyl lactate. Layer-3 atom novelty score = 3.042 (τ_{95} = 5.296), classifying the compound as in domain

Nonane-1-thiol

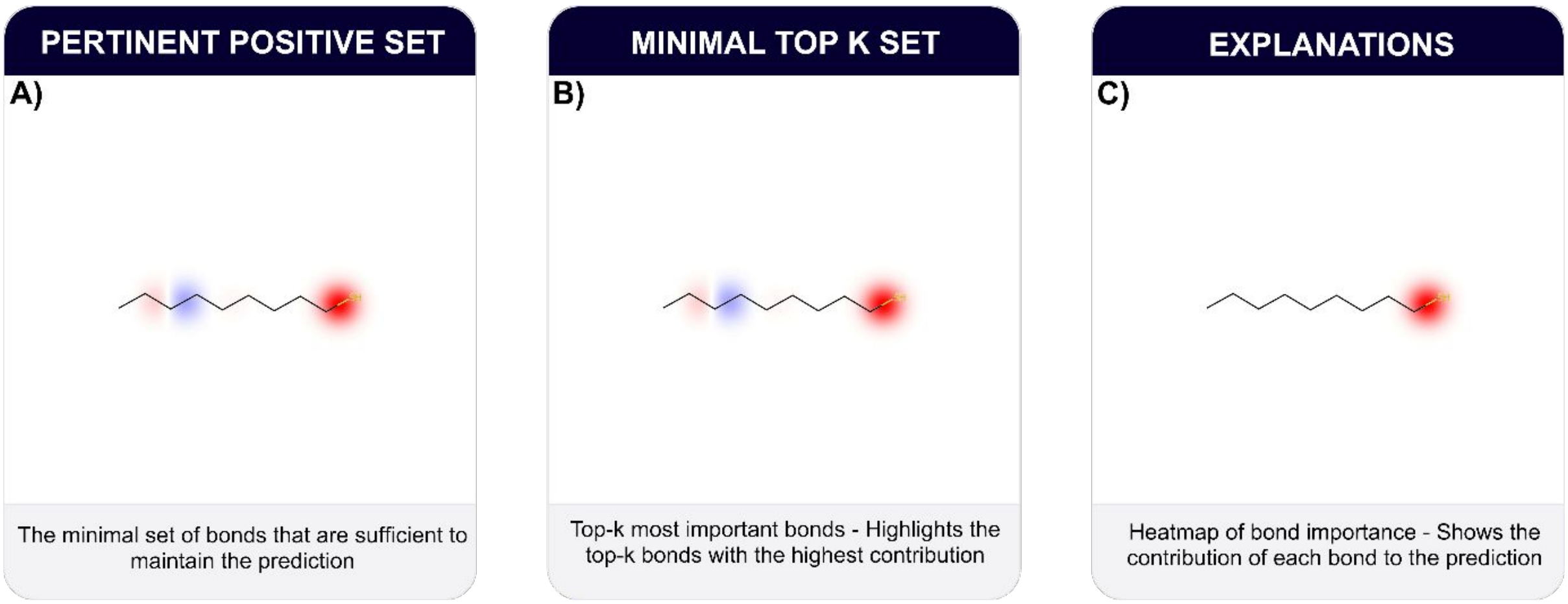

Figure S6 - EdgeSHAPer explanations for nonane-1-thiol

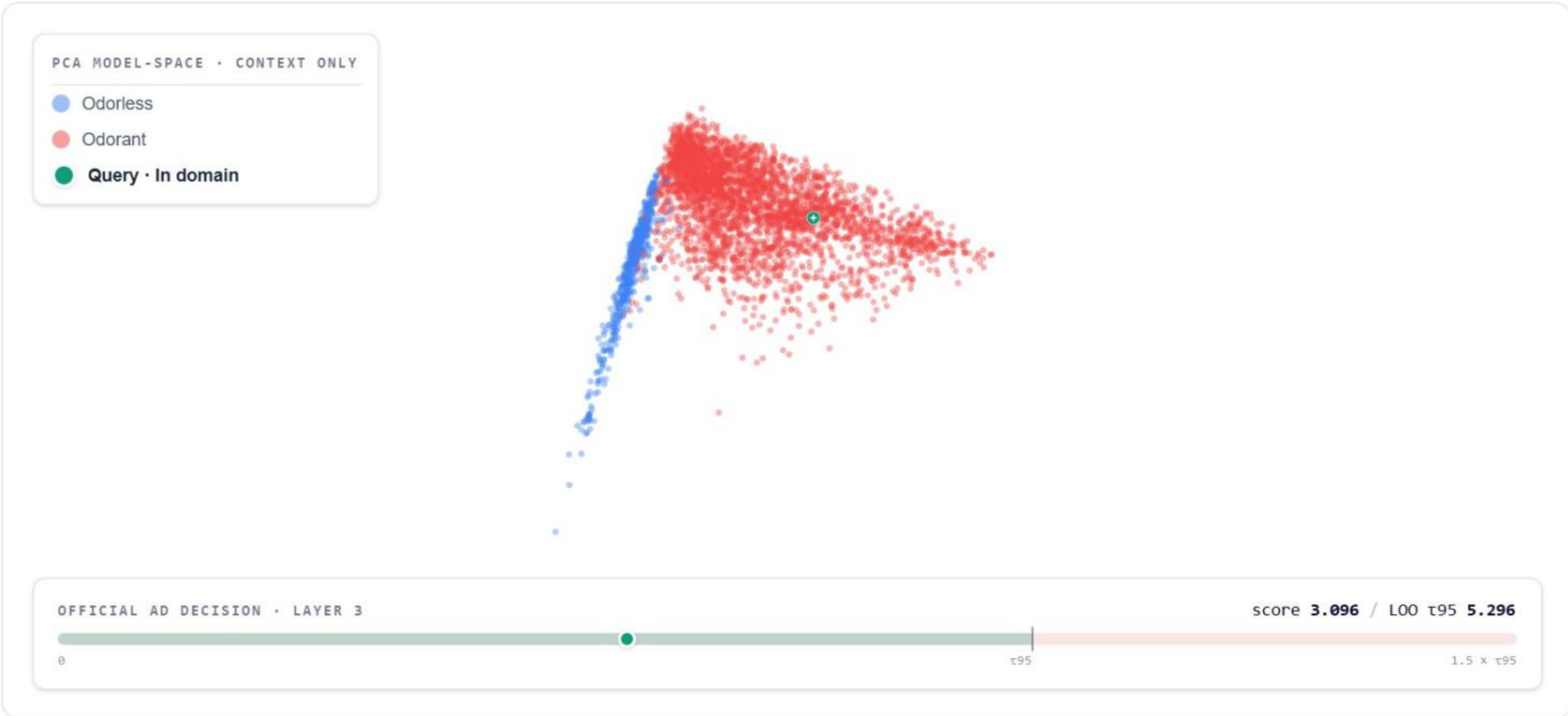

Figure S7 - Applicability domain assessment for nonane-1-thiol. Layer-3 atom novelty score = 3.096 (τ_{95} = 5.296), classifying the compound as in domain

Geraniol

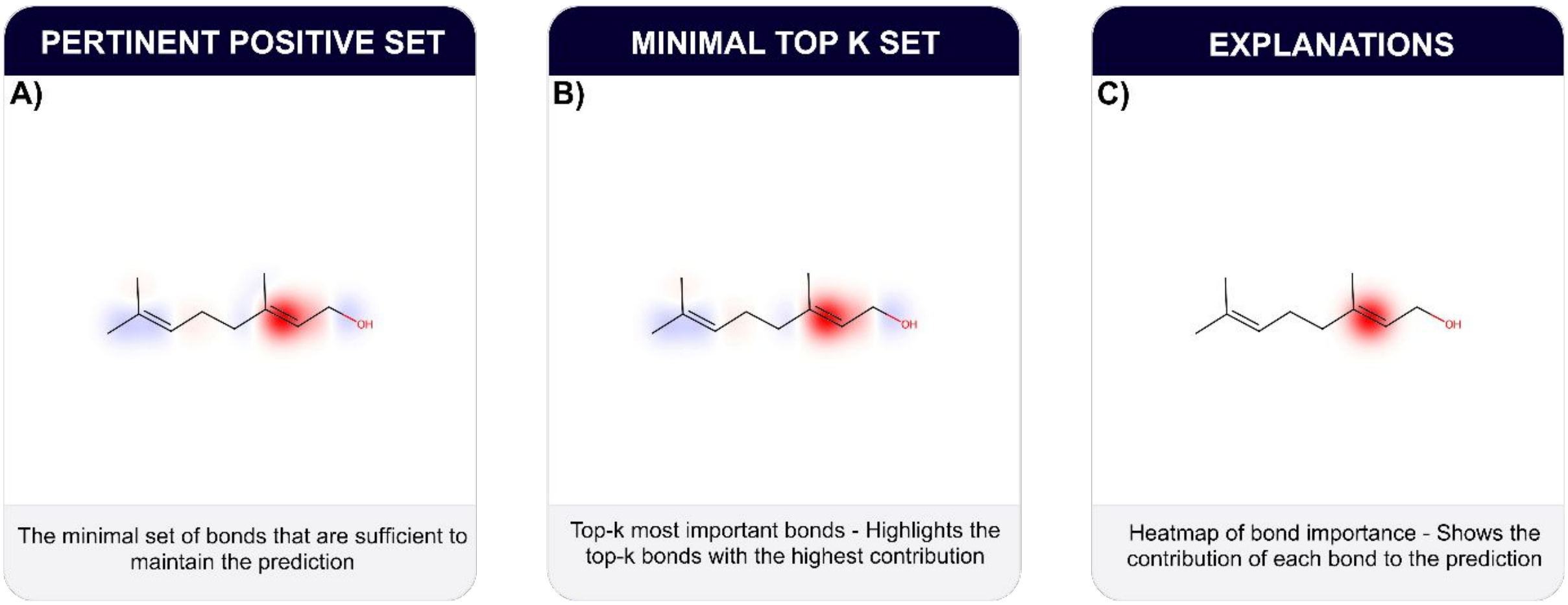

Figure S8 - EdgeSHAPer explanations for Geraniol

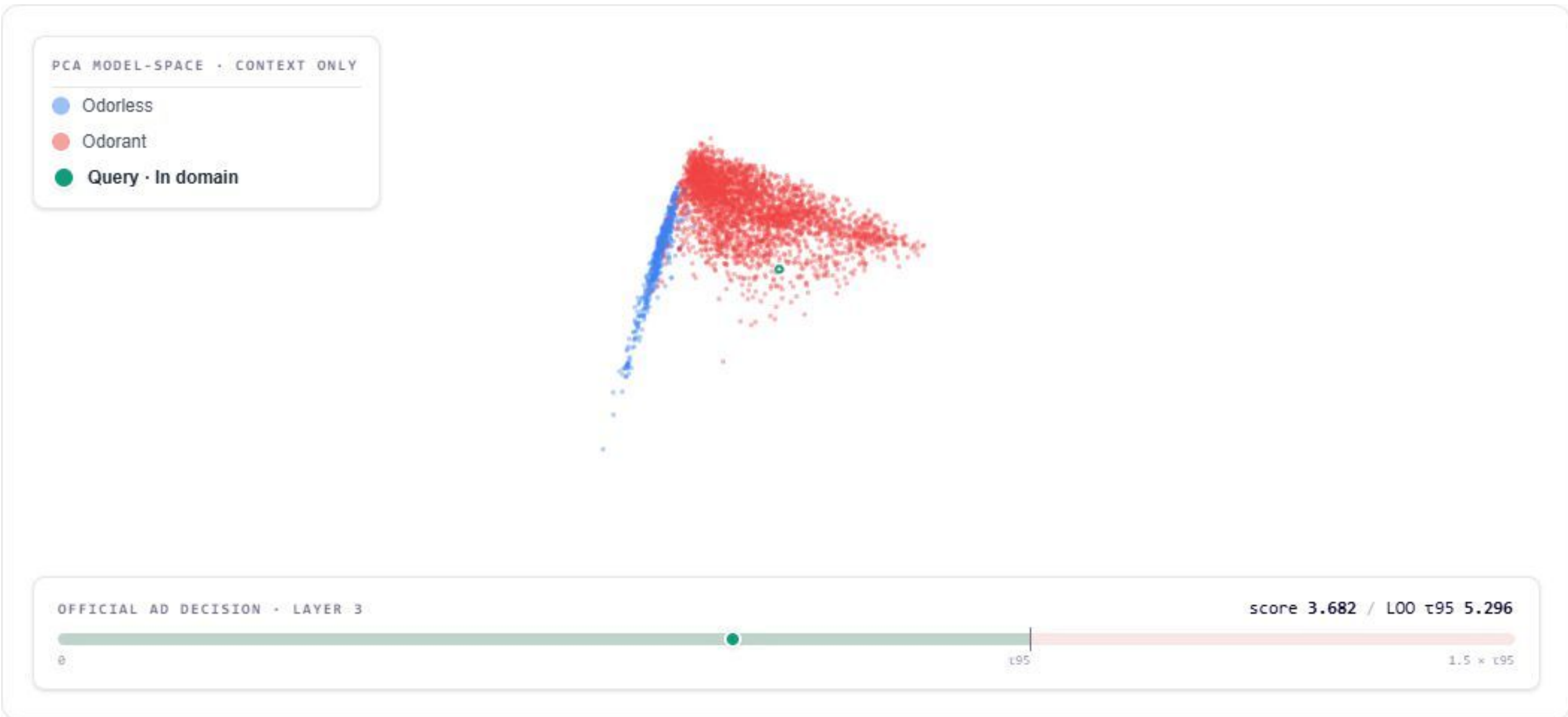

Figure S9 - Applicability domain assessment for geraniol. Layer-3 atom novelty score = 3.682 (τ_{95} = 5.296), classifying the compound as in domain

Limonene

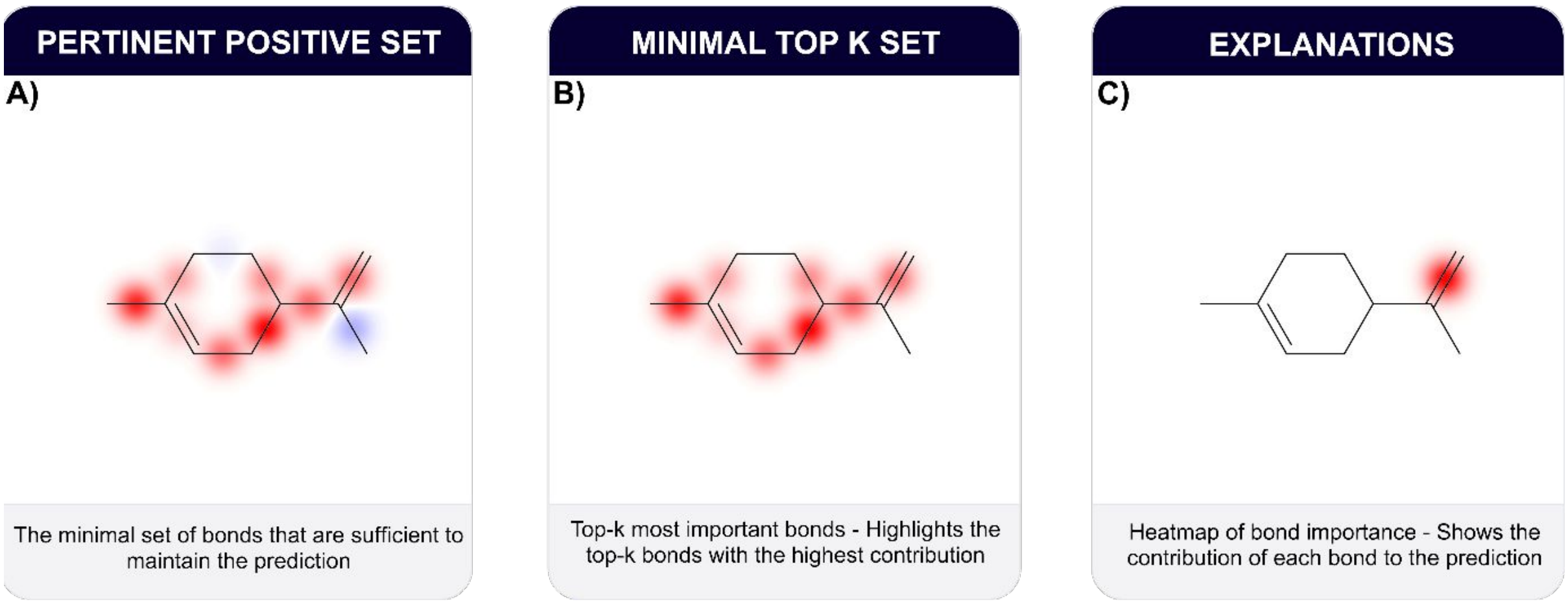

Figure S10 - EdgeSHAPer explanations for limonene

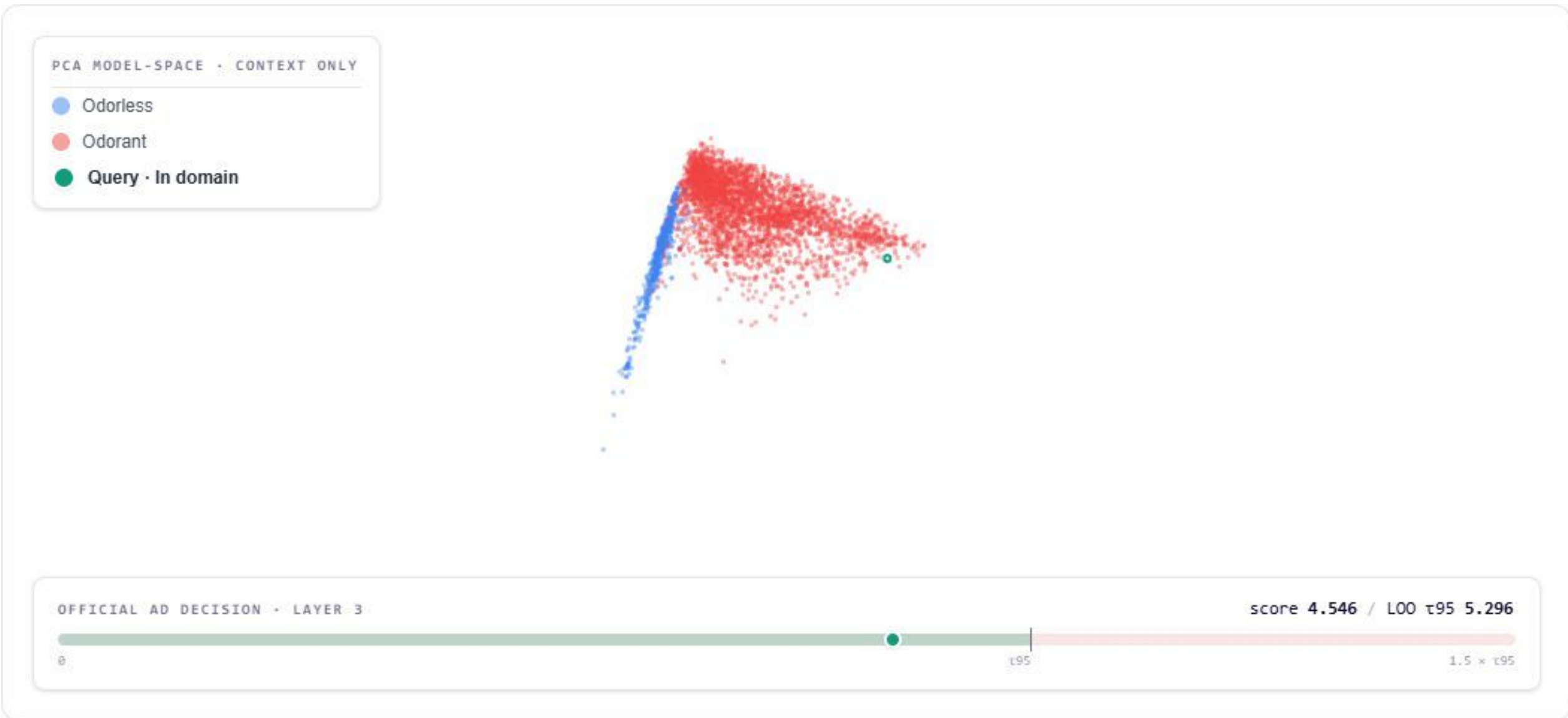

Figure S11 - Applicability domain assessment for limonene. Layer-3 atom novelty score = 4.546 (τ_{95} = 5.296), classifying the compound as in domain.

Monomenthyl succinate

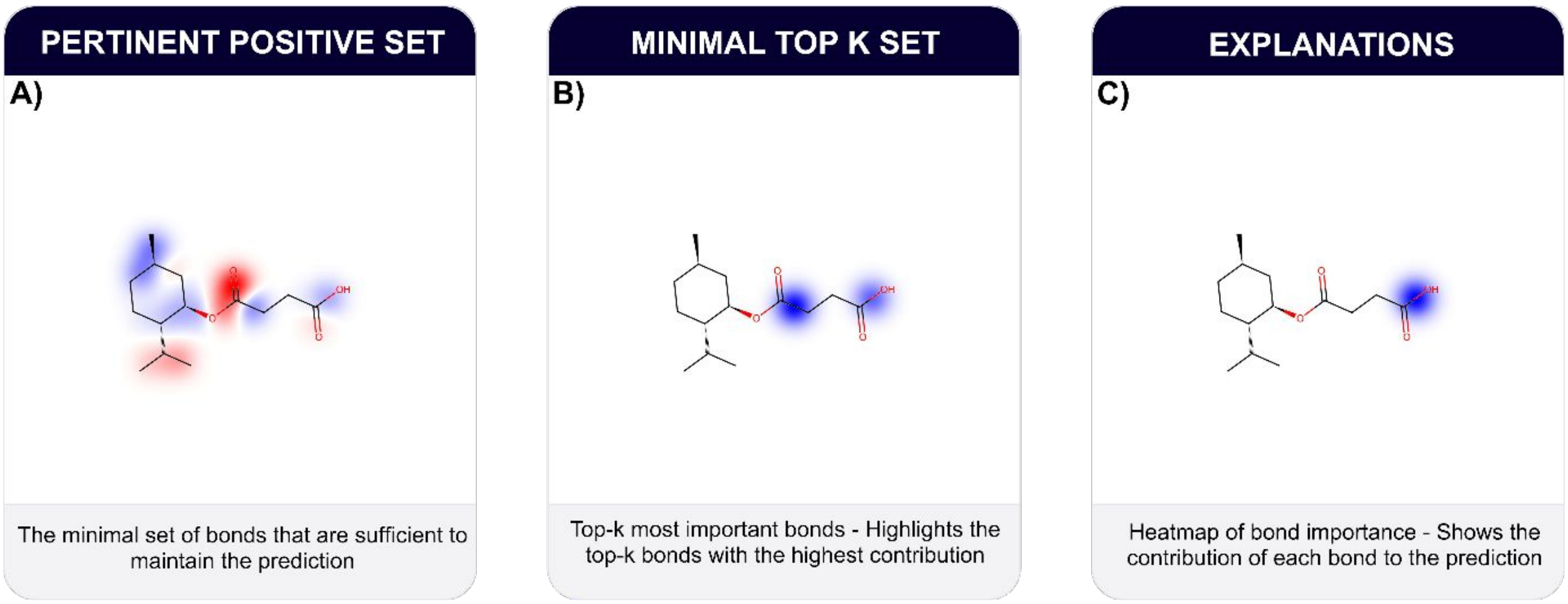

Figure S12 - EdgeSHAPer explanations for monomenthyl succinate.

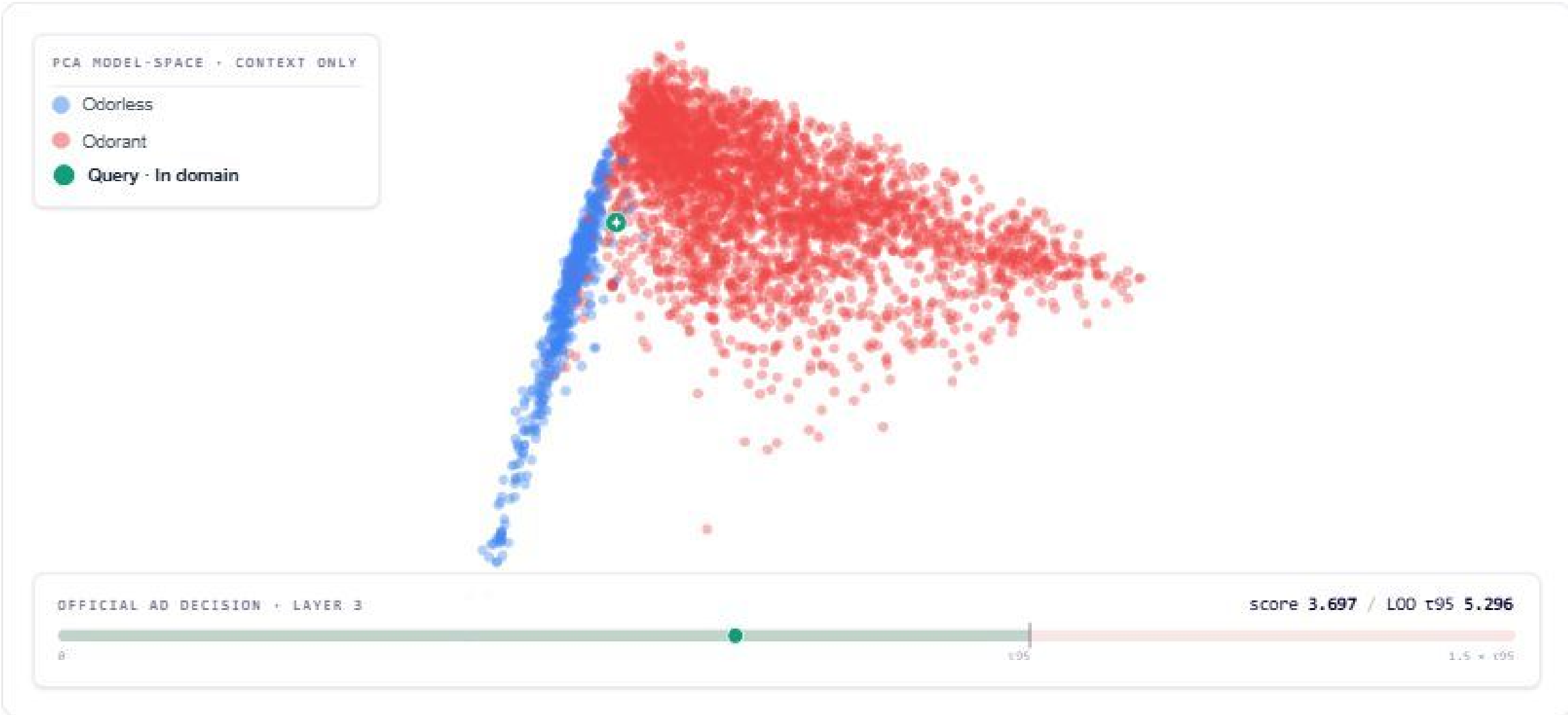

Figure S13 - Applicability domain assessment for monomenthyl succinate. Layer-3 atom novelty score = 3.697 (τ_{95} = 5.296), classifying the compound as in domain.
